# Non-coding RNAs: The Neuroinflammatory Regulators in Neurodegenerative Diseases

**DOI:** 10.3389/fneur.2022.929290

**Published:** 2022-08-12

**Authors:** Hao Jiang, Ying Zhang, Juan Yue, Yuchen Shi, Bo Xiao, Wenbiao Xiao, Zhaohui Luo

**Affiliations:** ^1^Department of Neurology, Xiangya Hospital, Central South University, Changsha, China; ^2^Xiangya School of Medicine, Central South University, Changsha, China; ^3^National Clinical Research Center for Geriatric Disorders, Xiangya Hospital, Central South University, Changsha, China; ^4^Clinical Research Center for Epileptic Disease of Hunan Province, Central South University, Changsha, China; ^5^Department of Geriatrics, The Second Xiangya Hospital of Central South University, Changsha, China

**Keywords:** ncRNA, neuroinflammation, neurodegenerative diseases, microglia, NLRP3, oxidative stress

## Abstract

As a common indication of nervous system diseases, neuroinflammation has attracted more and more attention, especially in the process of a variety of neurodegenerative diseases, such as Alzheimer's disease and Parkinson's disease. Two types of non-coding RNAs (ncRNAs) are widely involved in the process of neuroinflammation in neurodegenerative diseases, namely long non-coding RNAs (lncRNAs) and microRNAs (miRNAs). However, no research has systematically summarized that lncRNAs and miRNAs regulate neurodegenerative diseases through neuroinflammatory mechanisms. In this study, we summarize four main mechanisms of lncRNAs and miRNAs involved in neuroinflammation in neurodegenerative diseases, including the imbalance between proinflammatory and neuroprotective cells in microglia and astrocytes, NLRP3 inflammasome, oxidative stress, and mitochondrial dysfunction, and inflammatory mediators. We hope to clarify the regulatory mechanism of lncRNAs and miRNAs in neurodegenerative diseases and provide new insights into the etiological treatment of neurodegenerative diseases from the perspective of neuroinflammation.

## Introduction

Neuroinflammation is defined as an inflammatory response in the nervous system, including the brain and spinal cord, mediated by glial cells resident in the central nervous system, such as microglia and astrocytes, inflammatory mediators such as cytokines, chemokines, and reactive oxygen species, and second messengers produced by endothelial cells and immune cells migrated from the periphery (1). As an important immune activity, neuroinflammation takes part in promoting the repair of injured tissue and neuroprotection under normal physiological conditions (2). However, over-activated glial cells produce a large number of cytokines, give rise to strong inflammatory reactions and aggravate tissue injury, induce nerve degeneration, lead to the loss of the blood-brain barrier (BBB) and local infiltration of peripheral immune cells (3), resulting in immune, physiological, biochemical, psychological and other various effects (1).

Neurodegenerative diseases are incurable and debilitating conditions (4), which are characterized by the progressive deterioration of neuronal function attributed to the degeneration of synapses, axons, and ultimately the death of nerve cells (4, 5). This causes diverse problems with memory and cognitive, movement (called ataxias), or the ability to move, speak and breathe (4). In the central nervous system, the survival rate of neurons decreases, while the death of neurons increases, resulting in structural and functional abnormalities of the central nervous system. Classic neurodegenerative diseases include Alzheimer's disease (AD), Parkinson's disease (PD), and Amyotrophic Lateral Sclerosis (ALS). However, the acquired knowledge in respect of the pathophysiology of neurodegenerative diseases is limited and sometimes controversial. Current views include neuroinflammation, metabolic stress, and abnormal protein aggregates (6, 7). Some studies show that the related changes caused by neuroinflammation may promote the occurrence and development of neurodegenerative diseases (8). Neurodegeneration is mediated by inflammation and neurotoxic mediators, such as the elevation and activation of interleukin-1β (IL-1β), IL-6, tumor necrosis factor-α (TNF-α), granulocyte-macrophage colony-stimulating factor (GM-CSF), and nuclear factor kappa-B (NF-κB). Activated microglia, astrocytes, neurons, T cells, and mast cells release these inflammatory mediators and mediate neuroinflammation and neurodegeneration in a malignant way (3).

As the most important neurodegenerative disease, according to the World Alzheimer's Disease report, in 2019 there were more than 50 million AD patients worldwide, and the total number is expected to triple by 2050 (9). Neurodegenerative diseases seriously damage the quality of life of patients and bring heavy economic, medical, and social burdens. Yet, so far, traditional symptomatic treatment can only delay the progress of the disease, but not fundamentally cure such diseases. Therefore, it is imperative to find new treatments.

Genome-wide transcriptome analysis showed that most of the human transcriptional genome are non-coding RNAs (non-coding RNAs, ncRNAs) (10). Non-coding RNAs also include two types: short-chain ncRNAs (<200 nt) (11) and long-chain ncRNAs (long non-coding RNAs, lncRNAs, >200 nt) (12). With the deepening of research, it has been found that ncRNAs play an important role in the regulation of basic life processes such as cell proliferation, differentiation, transcription, post-transcriptional modification, and apoptosis (13). At present, in the field of neuroinflammation, the focus of ncRNAs research often concentrates on miRNAs and lncRNAs (14–17).

As an endogenous translation inhibitor, miRNAs act mainly by complementary pairing with the bases of the 3'untranslated region of the targeted transcript (18), resulting in its degradation or translation inhibition (19). There is evidence that extracellular vesicles (EVs) play a key role in intercellular communication by transporting mRNAs, miRNAs, and proteins, and in the Central Nervous System (CNS). This intercellular communication is particularly important for neuronal support and protection (especially during senescence), inflammation control, and clearance of infectious factors (20). In their study, Iyer et al. found that the expression of miR-146a was induced by IL-1β, while the miR-146a mimic could reduce the mRNA level of IL-6 and COX-2 (the two main inflammatory molecules induced by IL-1β) in cultured human glial cells, indicating that it plays an important role in human chronic inflammation-related nervous system diseases (21).

LncRNAs are a general term for a class of ncRNAs whose transcripts are longer than 200 nt. Their functions are highly diversified and still being studied and explored (22). In mammalian cells, lncRNAs play a role in all aspects of gene expression through different mechanisms: at the transcriptional regulation level, lncRNAs can affect chromatin tissue, formation of nuclear spot, and activity of RNA polymerase II; at the post-transcriptional regulation level, lncRNAs can regulate pre-mRNA splicing, accelerate or prevent the decay of mRNAs, inhibit or activate the translation of mRNAs; and at the level of post-translational regulation, lncRNAs can form scaffold assembly functional ribonucleoprotein complexes and affect the stability of proteins (23, 24). Moreover, lncRNAs can also act as an inhibitory regulator of miRNAs and target the expression of miRNAs (22). In the process of immune regulation, it has been suggested that lncRNAs play a role by directly or indirectly affecting a variety of effector proteins, such as transcription factors, acetylase, protein kinase, and phosphatase (25). At present, more and more studies have expounded on the role of lncRNAs in neuroinflammation. Han et al. speculated that lncH19 up-regulates the latency of temporal lobe epilepsy in rats and participates in the inflammatory response of epilepsy through bioinformatics analysis. Detection of pro-inflammatory cytokines released by activated glial cells found that the overexpression of H19 can stimulate the release of IL-1β, IL-6, and TNF-α in the hippocampal CA3 subdomain (26). Liu et al. showed that lncRNA Gm13568 interacts with histone acetyltransferase (HAT) CBP/P300 to promote the production of inflammatory cytokines and chemokines in activated astrocytes epigenetically, then promotes the infiltration of inflammatory cells and demyelinating lesion in experimental autoimmune encephalomyelitis (EAE) mice (27).

With research progress of ncRNAs in the field of neuroinflammation, it provides us with new ideas for the etiological treatment of neurodegenerative diseases. This study will focus on the four main mechanisms (imbalance of the pro-inflammatory and anti-inflammatory ratio of microglia and astrocytes; NLRP3 inflammatory bodies; inflammatory mediators; and oxidative stress and mitochondrial dysfunction) involved in neurodegenerative diseases through the regulation of neuritis by ncRNAs. This study will also describe how ncRNAs participate in the occurrence and development of neurodegenerative diseases by regulating neuroinflammatory responses and provide new ideas for the etiological treatment of neurodegenerative diseases.

## The Imbalance Between Proinflammatory and Neuroprotective Cells in Microglia and Astrocytes

**Figure 1:**
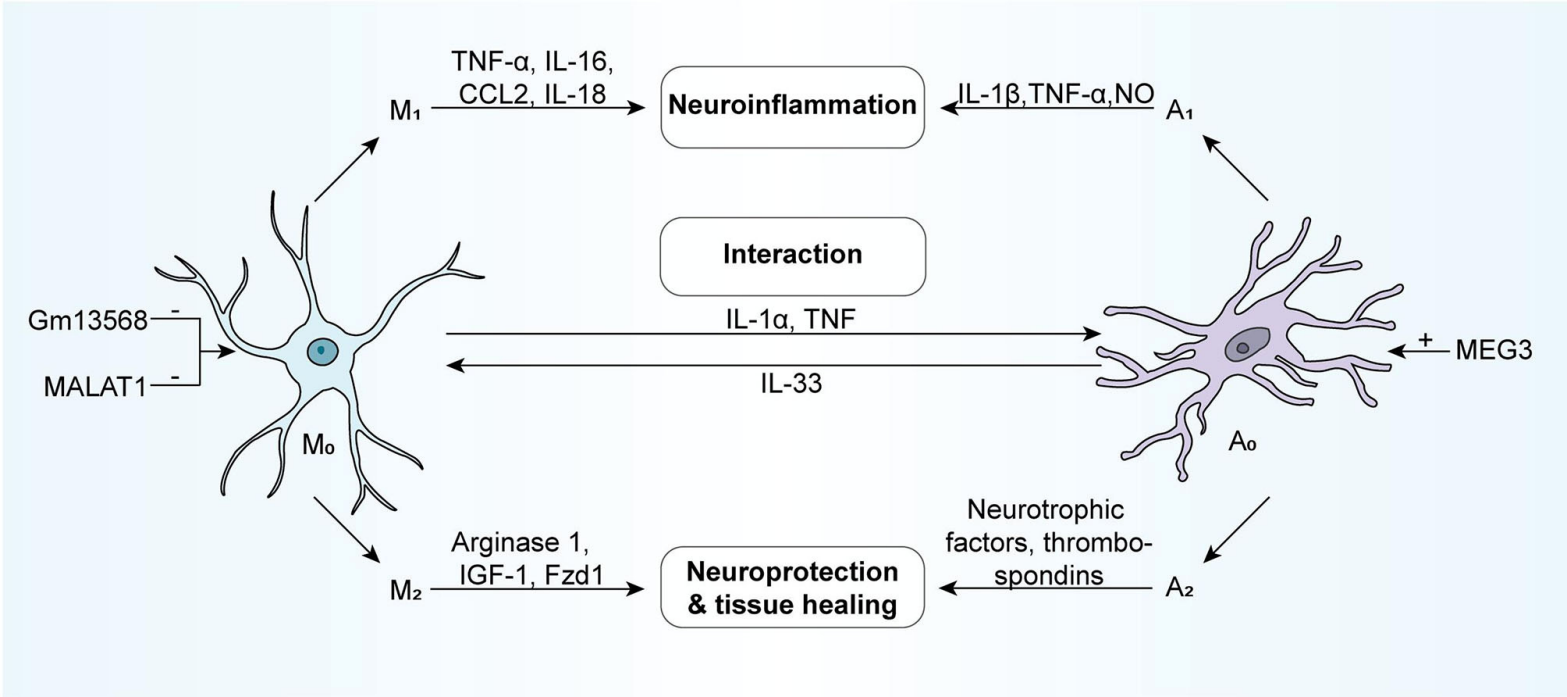
NcRNAs can involve in the regulatory processes of the activation of microglia and astrocyte. Generally, microglia and astrocytes respond to external stimuli as a whole, functionally restricting each other and coordinating with one another. According to activation status, they are usually divided into two phenotypes: the neurotoxic phenotype (M1/A1) and the neuroprotective phenotype (M2/A2). The neurotoxic microglia, which secretes pro-inflammatory cytokines (e.g., TNF-α, IL-16, and so forth) and chemokines (e.g., CCL2, IL-18, and so forth), participate in neuroinflammation. While the neuroprotective microglia could release Arginase 1, IGF-1, Fzd1, and so forth, contributing to neuroprotection and tissue healing. The neurotoxic astrocytes induce the production of pro-inflammatory factors such as IL-1β, TNF-α, NO, and so forth, while neuroprotective astrocytes secrete neurotrophic factors and thrombospondins in large quantities. Astrocytes and microglia can directly interact with one another via molecules like IL1a, TNF, and IL-33. M1, neurotoxic microglia; A1, neurotoxic astrocyte; M2, neuroprotective microglia; A2, neuroprotective astrocyte; IGF-1, insulin-like growth factor 1; Fzd1, Frizzled-1.

Microglia and astrocytes exist widely in the central nervous system. Some studies show that microglia and astrocytes can communicate with each other through IL-1α, TNF and other communication molecules (28), and their functions were restricted and coordinated. This relationship is especially significant in pathological conditions, and when the brain is disturbed by external interference, it often responds as a whole (28). According to the activation state, both of them can be divided into two phenotypes-neurotoxicity (M1/A1) and neuroprotection (M2/A2). Neurotoxic microglia participate in neuroinflammation by secreting pro-inflammatory cytokines (TNF- α, IL-16, etc.) and chemokines (CCL2, IL-18, etc.), while neuroprotective microglia release arginase 1, IGF-1, and Fzd1 involved in neuroprotection and tissue healing (29, 30). Pro-inflammatory reactive astrocytes induce the production of pro-inflammatory factors such as IL-1 β, TNF- α, and NO, while neuroprotective astrocytes secrete a large number of neurotrophic factors and thrombospondin (Figure 1).

### ncRNAs Which Act on a Variety of Neurodegenerative Diseases

#### LncRNA MALAT1

Studies show that Nrf2 can regulate the activation of microglia (31), which is highly expressed in the brain of PD model mice induced by MPTP and microglia induced by LPS/ATP. MALAT1 inhibits the expression of Nrf2 by epigenetic means, thus promoting microglial inflammation, leading to M1/M2 imbalance and aggravating nerve damage in PD (32).

In patients with MS, the expression of MALAT1 is significantly down-regulated. Researchers suggest that the down-regulation of MALAT1 may enhance the differentiation of M1 cells, resulting in an imbalance in the proportion of microglia M1/M2 and participating in the neuroinflammatory response of MS (33).

#### LncRNA-GAS5

The results of PD-related experiments showed that there is a negative correlation between the expression of lncRNA GAS5 and miR-223-3p in the PD mouse model. Bioinformatics analysis and double luciferase reporter gene analysis showed that there is a direct targeting relationship between GAS5 and miR-223-3p (34). It is speculated that GAS5 may act to down-regulate the expression of miR-223-3p. By detecting the inflammatory response of microglia activated by LPS, it is found that the knockdown of GAS5 can inhibit the inflammation of microglia. The overexpression of miR-223-3p has a similar result. As PD progresses, lncRNA-GAS5 can enhance the inflammation of microglia and antagonize the anti-inflammatory effect of miR-223-3p (34).

Moreover, in the study of multiple sclerosis (MS), lncRNA-GAS5 inhibits the transcription of TRF4 (the key gene controlling M2 cell polarization) by recruiting PRC2 and increasing the expression of TNF- α and IL-1 β, which are cell markers of M1, thus inhibiting polarization of microglia M2 of and promoting polarization of M1. Finally, it leads to the imbalance of M1/M2 proportion of microglia and participates in the neuroinflammatory injury of MS (35).

#### miR-155

Studies show that miR-155 is overexpressed in superoxide dismutase I (SOD1) mouse models and sporadic and familial ALS patients. A variety of proinflammatory genes in microglia were significantly up-regulated and the proportion of proinflammatory cells was also up-regulated. The gene ablation experiment of miR-155 reversed the high expression of pro-inflammatory genes in microglia of the SOD1 mouse model and restored the phagocytosis of microglia, delaying the onset of disease and prolonging the survival time of the SOD1 mouse model (36). It is speculated that miR-155 may participate in the neuroinflammatory injury of ALS by up-regulating the proportion of pro-inflammatory microglia and disrupting the balance of M1/M2.

In the study of curcumin, it was found that curcumin can treat AD by down-regulating the expression of miR-155 and inhibiting the activation of microglia and astrocytes (37).

### ncRNAs Which Act on a Particular Disease

#### Down-Regulate the Proportion of Microglia M1 Phenotype by Inhibiting lncRNA HOXA11-AS From Passing miR-124-3p–FSTL1/NF-κB Axis (PD)

Studies show that lncRNA HOXA11-AS enhanced MPTP-mediated SH-SY5Y neuronal damage and participated in LPS-induced microglial activation and the proportion of pro-inflammatory phenotypes increased (38). As the target of lncRNA HOXA11-AS, miR-124-3p has the opposite effect with HOXA11-AS in neuroinflammation and reduces inflammatory injury. In addition, there is a negative correlation between the expression of FSTL1 and miR-134-3p. Besides, inhibition of NF-κB can attenuate neuroinflammation caused by the high expression of HOXA11 (38). In the end, the researchers speculated that the inhibition of lncRNA HOXA11-AS may down-regulate the proportion of microglia phenotype M1 and inhibit neuroinflammation by targeting the miR-124-3p–FSTL1/NF-κB axis.

#### LncRNA-p21 Induces Microglial Activation by Acting on the miR-181/PKC-δ Regulatory Pathway (PD)

Studies show that lncRNA-p21 plays an important role in the activation of microglia through the p53 pathway. At the same time, lncRNA-p21 targets miR-181. The interaction between lncRNA-p21 and miR-181 may induce the degradation of lncRNA-p21 and weaken the activation of microglia (39). In addition, as a specific target of miR-181, the expression of PKC-δ decreased with the up-regulation of miR-181. Studies show that there is also an interaction between lncRNA-p21 and PKC- δ. The overexpression of lncRNA-p21 up-regulated the expression of PKC-δ, and they were eliminated by the ectopic expression of miR-181. LncRNA-p21 and miR-181 family / PKC-δ formed a double-negative feedback loop, which cooperatively promoted the activation of microglia (39).

#### Down-Regulates the Proportion of Proinflammatory Astrocytes by Inhibiting LncRNA Gm13568 From Passing the Notch Signal Pathway (MS)

A variety of abnormally expressed lncRNAs have been identified in MS studies, among which lncRNA-Gm13568 may target the Notch signal pathway (27), which has been proved to be closely related to neurodegenerative diseases (40) and may lead to inflammatory activation and proliferation of astrocytes (41). Through experimental evaluation, the researchers have proved that the knockdown of Gm13568 significantly inhibited the expression of Notch1 and reduced the proportion of reactive astrocytes, and the production of inflammatory factors was significantly reduced (27). Therefore, inhibition of lncRNA-Gm13568 can restore the balance between pro-inflammatory and anti-inflammatory astrocytes and reduce the injury of nerve inflammation in MS by acting on the Notch pathway.

#### LncRNA-SNHG1 Targets to Regulate miR-7 and Then Up-Regulate the Proportion of Pro-inflammatory Microglia (PD)

The experiment showed that the expression of microglial activation markers in PD animal models with high expression of SNHG1 is significantly increased. The expression of SNHG1 and miR-7 are negatively correlated, and miR-7 can inhibit the activation of microglia to pro-inflammatory phenotype (42). In short, lncRNA-SNHG1 down-regulates the expression of miR-7, up-regulates the proportion of pro-inflammatory microglia, and then participates in neuroinflammatory injury.

### Miscellaneous

Some ncRNAs regulate neuroinflammation by regulating the pro-inflammatory and anti-inflammatory ratios of microglia and astrocytes. For example, lncRNA-MEG3 in AD reduces the proportion of pro-inflammatory astrocytes to correct the imbalance of A1/A2 (43). After incubation with let-7, a large number of M1 microglia appear, which aggravates neuroinflammatory damage (44); miR-146a changes the polarization direction of microglia and improves astrocyte inflammation to reduce neuroinflammation (45, 46). In addition, miR-124 is the key regulator to maintaining the quiescent state of microglia. The low expression of miR-124 in MS patients leads to the abnormal increase of pro-inflammatory microglia, and the imbalance of the M1/M2 ratio aggravates the nerve inflammatory injury (47). In the ALS model, miR-218 is highly expressed and absorbed by astrocytes to promote its transformation into a pro-inflammatory phenotype. Knocking down miR-218 can reduce the proliferation of astrocytes and inhibit neuroinflammation (48).

## NLRP3 Inflammasome

NLRP3 inflammasome is the most widely studied inflammasome, consisting of nucleotide-binding oligomeric structures, leucine-rich repetitive structures, and pyrin structural domains (49). It is extensively involved in the development of neurodegenerative diseases (50–52). Research shows that the activation of NLRP3 requires a dual signaling action of initiation and activation (53). Activation of the NLRP3 inflammasome leads to the activation of caspase-1, which mediates the production of pro-inflammatory cytokines such as IL-1β and IL-18, and then initiates the inflammatory response (54, 55).

IL-1β allows peripheral immune cells to recruit and infiltrate the central nervous system by regulating the integrity of the BBB and increasing the expression of chemokines (56, 57). Besides, IL-1β stimulates inflammatory activation of microglia and astrocytes, causes dysregulation of the pro-inflammatory and anti-inflammatory ratio (M1/M2), and promotes the development of neuroinflammation (58).

IL-18 activates microglia, up-regulates caspase-1 expression, and increases matrix metalloproteinase as well as pro-inflammatory cytokine production, followed by participation in a neuroinflammatory activity (59).

**Figure 2:**
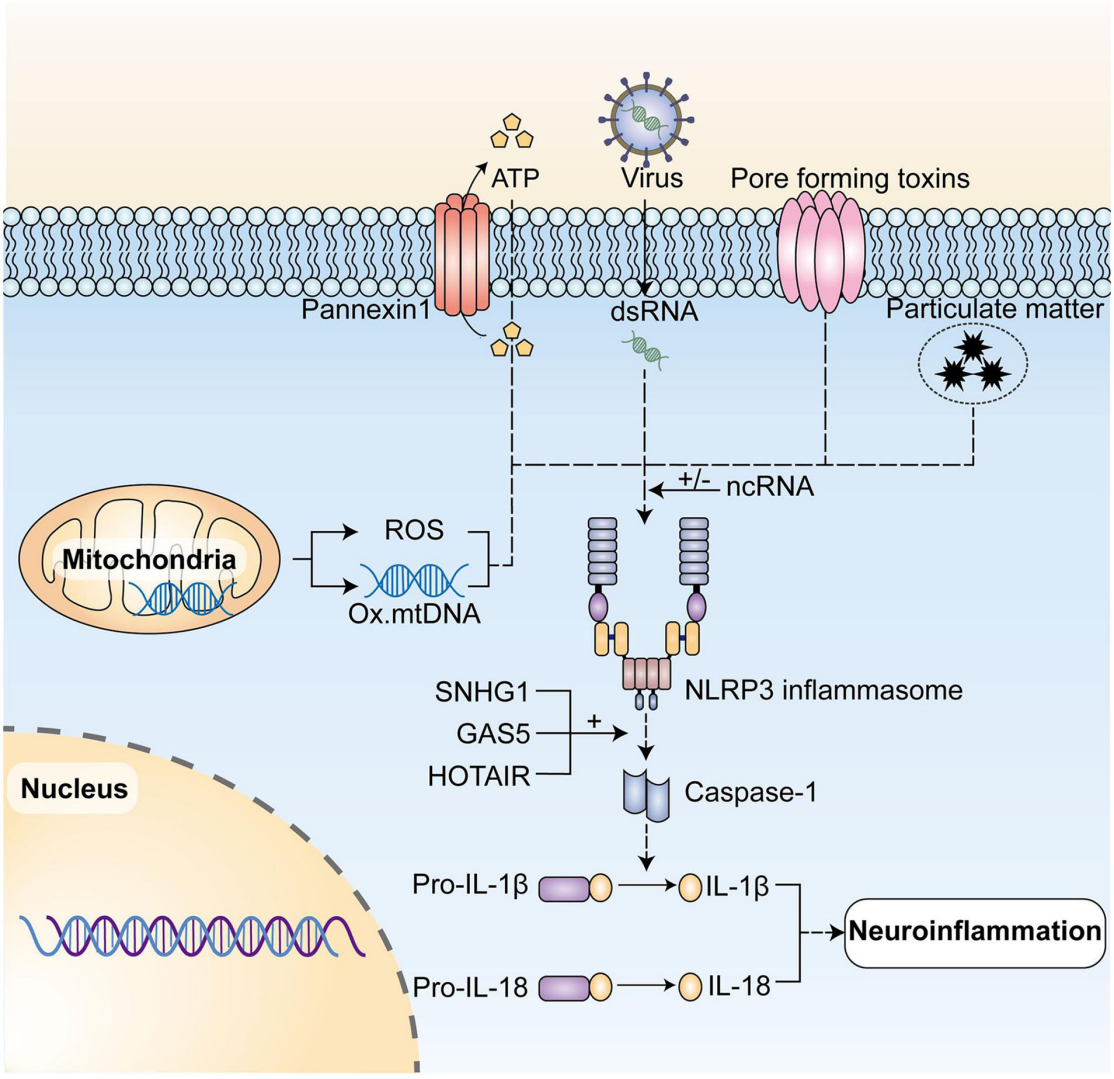
NcRNAs play a regulatory role in the activation of NLRP3 inflammasome and its post-translational modification. The NLRP3 inflammasome initiates, activates, and then posttranslational modifications under the action of initiating signals, including dsRNAs of RNA viruses, and activation signals, such as extracellular ATP, pore-forming toxins, particulate matter, mtDNA, and ROS produced by mitochondria and so forth, leading to the caspase-1 activation. In turn, it mediates the production of pro-inflammatory cytokines such as IL-1β and IL-18, and ultimately initiates neuroinflammation. NLRP3, NLR family pyrin domain containing 3; mtDNA, mitochondrial DNA; ROS, reactive oxygen species. LncRNA SNHG1, lncRNA GAS5, and lncRNA HOTAIR can play a regulatory role by up-regulating NLRP3 expression. NLRP3, NLR family pyrin domain containing 3; mtDNA, mitochondrial DNA; ROS, reactive oxygen species.

Recent studies demonstrated the important contribution of NLRP3 to a variety of neurodegenerative diseases, with overexpression of NLRP3 leading to overproduction of IL-1β, IL-18 pro-inflammatory cytokines, and concomitant chronic neuroinflammation throughout the development and progression of neurodegenerative diseases (60). These studies have suggested new ideas on how to down-regulate NLRP3 expression to inhibit neuroinflammation, then treat neurodegenerative pathologies and ncRNAs associated with NLRP3 regulation (Figure 2).

### ncRNAs Which Act on a Variety of Neurodegenerative Diseases

#### LncRNA MALAT1

It has been shown previously that lncRNA MALAT1 is involved in neuroinflammation in MS and PD by regulating the pro-inflammatory and anti-inflammatory ratio of microglia cells. In addition, the study showed that high expression of MALAT1 is significantly associated with NLRP3 activation in a mouse PD model established by MPTP, while knockdown of MALAT1 weakens inflammasome activation. MALAT1 may regulate inflammasome by inhibiting the expression of the antioxidant transcription factor Nrf2 (32).

#### Alu RNA

Alu is the most abundant potential source of ncRNAs in the human genome and has been shown to activate inflammatory pathways and apoptosis in non-neural cells such as the retinal pigment epithelium (RPE). Alu RNA plays an important role in iron-dependent degeneration of the RPE, which also occurs with the help of inflammasome NLRP3 (61). Whereas, accumulation of Alu RNA was observed in a variety of degenerative brain diseases (62), researchers speculated that Alu RNA may be involved in the neuroinflammatory process of multiple neurodegenerative diseases by regulating inflammasome NLRP3 expression (63).

#### LncRNA 4344 Targets miR-138-5p and Up-Regulates NLRP3 Expression

In the study of neurological disorders related to cognitive disorders such as AD and PD, researchers found that lncRNA 4344 was highly expressed in the hippocampus of rats with cognitive disorders, and a significant positive correlation between the expression of lncRNA 4344 and NLRP3 was obtained by correlation analysis (64). Structural prediction, as well as correlation analysis, showed that lncRNA 4344 expression levels were significantly and negatively correlated with miR-138-5p expression levels, and NLRP3 was a direct downstream target of miR-138-5p and directly negatively bound to miR-138-5p (64). In summary, lncRNA 4344 is involved in neuroinflammatory processes in neurological disorders associated with cognitive disorders by competitively binding to miR-138-5p, thereby indirectly up-regulating the expression of inflammasome NLRP3.

#### LncRNA KCNQ1OT1 Targets miR-30e-3p and Up-Regulates NLRP3 Expression

Both KCNQ1OT1 and NLRP3 expression levels were significantly elevated in LPS-induced HMC3 cells. LncRNA KCNQ1OT1 inhibits the expression of miR-30e-3p and miR-30e inhibits NLRP3 expression thereby attenuating neuroinflammatory injury. lncRNA KCNQ1OT1 is involved in a variety of inflammatory-related neurological diseases by inhibiting miR-30e-3p and thereby up-regulating the NLRP3 expression (65).

### ncRNAs Which Act on a Particular Disease

#### LncRNA SNHG1 Targets miR-7 and Up-Regulates NLRP3 Expression (PD)

The experimental results showed that SNHG1 and NLRP3 share the same response element of miR-7 in the LPS-induced PD model. SNHG1 down-regulates miR-7 expression and miR-7 suppresses NLRP3 expression. Taken together, lncRNA SNHG1 is involved in neuroinflammatory injury in PD by down-regulating miR-7 expression and thus up-regulating NLRP3 expression (42).

#### LncRNA-GAS5 Targets miR-223-3p to Up-Regulate NLRP3 Expression (PD)

The possible targeting roles of lncRNA-GAS5 and miR-223-3p have been described previously. In addition, researchers found a binding relationship between miR-223-3p and NLRP3, with miR-223-3p overexpression significantly reducing NLRP3, ASC, and phosphorylated caspase1 levels. On the whole, these data suggest that GAS5 is involved in neuroinflammatory damage in the course of PD by down-regulating miR-223-3p expression and thereby up-regulating NLRP3 expression (34).

#### LncRNA-HOTAIR Up-Regulates NLRP3 Expression Through miR-326/ELAVL1 Regulatory Axis (PD)

In the PD mouse models, lncRNA-HOTAIR expression was significantly up-regulated, while knockdown of lncRNA-HOTAIR attenuated neuronal damage in PD, and it was hypothesized that lncRNA-HOTAIR is significantly associated with the pathological changes of PD (66). While lncRNA-HOTAIR down-regulated the expression of miR-326 by sponging it, ELAVL1, a direct target of miR-326, formed a lncRNA-HOTAIR-miR-326/ELAVL1 regulatory axis. Overexpression of lncRNA-HOTAIR or ELAVL1 significantly down-regulated miR-326, followed by up-regulation of NLRP3 expression, which then exacerbated neuroinflammatory injury in PD (66).

### Miscellaneous

In addition, lncRNA HOTTIP was found to enhance NLRP3 activation by targeting and inhibiting miR-615-3p in the PD model (67). lncRNA-SNHG14 was found to down-regulate miR-223-3p and then up-regulate NLRP3 expression in the AD study (68). While miR-212 attenuated neuroinflammation in AD rats by inhibiting SP1 expression to block BACE1-induced NLRP3 activation (69).

## Oxidative Stress and Mitochondrial Dysfunction

Many studies have found that oxidative stress and mitochondrial dysfunction may cause neuroinflammation, leading to most neurodegenerative pathologies, including amyotrophic lateral sclerosis, Alzheimer's disease, and Parkinson's disease (70, 71). It occurs when mitochondria produce an excess of ROS, which results in oxidative stress, ultimately leading to neuroinflammation (72).

In the physiological state, mitochondria produce mtRNA that encodes part of the structure of ATP, as well as the required protein (73). This process releases reactive oxygen species (ROS), and superoxide dismutase (SOD) in the cell removing ROS as well as other harmful substances from the cell. When mitochondria are dysfunctional, ROS causes mtRNA rearrangement and mutation, resulting in reduced ATP synthesis and intracellular ROS buildup (74), which in turn causes cellular oxidative stress (75) and initiates caspase-7 and caspase-3 into apoptosis (76). It has also been shown that the accumulation of intracellular ROS can also lead to the accumulation of more mutant mtRNAs (74). That is, mitochondrial dysfunction and oxidative stress in neuroinflammation is a mutually reinforcing process which eventually leads to apoptosis. When oxidative stress occurs, the levels of SOD, CAT, GSH-Px, and SIRT2 in neuronal cells are rapidly down-regulated, while tau protein phosphorylation is rapidly up-regulated, making it difficult to resist the intracellular accumulation of ROS thereby causing severe oxidative stress in neurons (77, 78).

Besides, the accumulation of ROS can also cause the expression of many inflammatory factors in activated cells, such as NF-κB, TNF-α, IL-1β, and IL-6. They are closely associated with neuroinflammation (79). Neuroinflammation and oxidative stress are interrelated and mutually involved in neurodegenerative diseases. Baek JY. et al. found that in neurodegenerative diseases, microglia are activated to produce and release ROS as well as inflammatory cytokines such as IL-1β and IL-6 to exert neurotoxic effects, thereby triggering oxidative stress and neuroinflammation (80). The mtROS produced by mitochondrial respiratory chain inhibition due to mitochondrial dysfunction can activate the NLRP3 inflammasome (81). Likewise, oxidation of newly synthesized mtDNA induced by toll-like receptors (TLR) signaling also promotes inflammasome NLRP3 activation (82).

**Figure 3:**
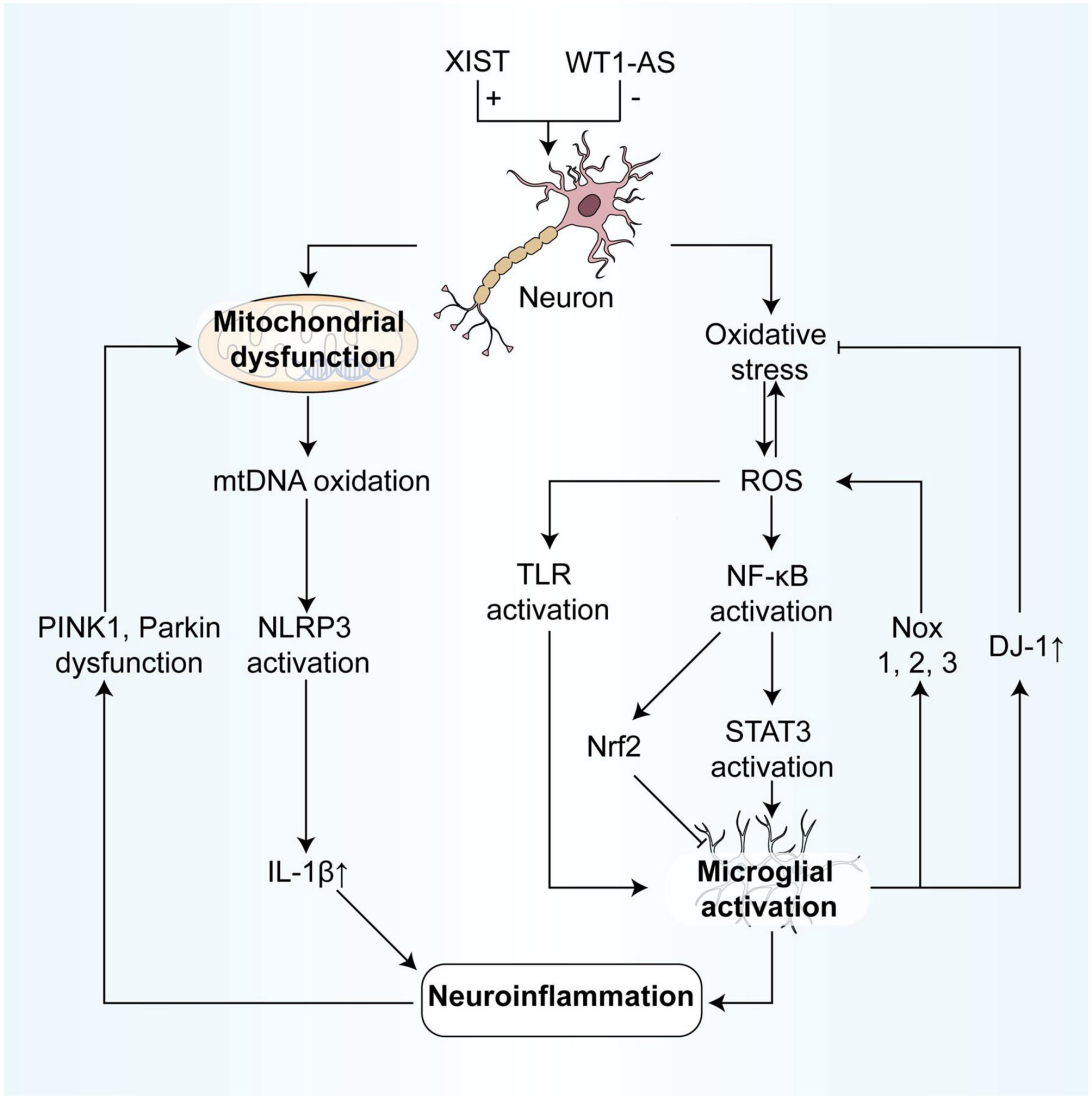
NcRNAs participate in the association between oxidative stress and neuroinflammation, regulating parts of the process. On the one hand, the process from oxidative stress to neuroinflammation includes: (1) ROS modifies and activates TLRs, resulting in the inflammatory response of microglia; (2) ROS degrades IκB and promotes NF-κB activation, which in turn activates STAT3, finally leading to microglia activation to participate in neuroinflammation. (3) Also, after NF-κB is activated by ROS, the activation of Nrf2 can inhibit the microglia activation, resulting in anti-inflammatory effects. (4) Mitochondrial oxidative mtDNA mediates the activation of the NLRP3 inflammasome, resulting in the production of IL-1β, and in turn, induces neuroinflammation. On the other hand, the effects of neuroinflammation on oxidative stress include: (1) Activated microglial cells can not only produce the large expression of NOX1, 2,3, inducing oxidative stress but also inhibit oxidative stress by expressing large quantities of DJ-1. (2) In neuroinflammation, the dysfunction of PINK 1 and Parkin leads to mitochondrial autophagy blockage, and then results in mitochondrial dysfunction and cell apoptosis. IκB, IkappaB kinase; NF-κB, nuclear factor kappa-light-chain-enhancer of activated B cells; Nrf2, Nuclear factor-erythroid factor 2-related factor 2; NOX, NADPH oxidase; PINK 1, PTEN-induced kinase 1.

Many articles have investigated how ncRNAs prevent neurodegenerative diseases by regulating oxidative stress and mitochondrial dysfunction. These studies provide therapeutic directions for the clinical treatment of neurodegenerative diseases (Figure 3).

### ncRNAs Which Act on a Variety of Neurodegenerative Diseases

#### miR-137/491

Dopamine accumulation in neuronal cells can trigger oxidative stress in neuronal cells, which can damage neurons and induce neurodegenerative disease (83). In neurodegenerative lesions, miR-137 targets the 3′ untranslated regions (UTR) of SLC6A3 mRNA, and miR-491 targets the variable number of tandem repeats (VNTR) sequence of the 3′ UTR of SLC6A3 mRNA to significantly reduce the level of SLC6A3 mRNA and down-regulate the expression of dopamine transporter (DAT) at the post-transcriptional level, thereby reducing the oxidative stress damage in neurons (84).

#### LncRNA H19

Feng M. et al. found that miR-130a-3p expression was up-regulated in the SH-SY5Y cell model, promoting apoptosis and brain injury, while lncRNA-H19, down-regulated miR-130a-3p expression thereby increasing DAPK1 content, reducing oxidative stress and protecting against brain injury (85).

#### LncRNA-NEAT1

The expression of miR-221-3p is increased in neurodegenerative diseases (86). LncRNA-NEAT1 inhibits miR-221-3p expression, then reduces the role of miR-221-3p and suppresses SIRT2 expression through post-transcriptional regulation (87). The SIRT2 inhibitor AGK2 induces vascular endothelial growth factor (VEGF) and HO-1 gene expression by downregulating SIRT2, then enhancing the resistance of neurons to oxidative stress (88). In addition, miR-486-3p, miR-8061, miR-376a-5p, miR-486-3p, miR-8061, and miR-376a-5p bind to different sites of 3'UTR of SIRT2 and inhibit the translation of SIRT2 (89, 90). This implies that the above miRNAs can be used as clinical targets to protect neurons by regulating SIRT2 content.

#### Multiple miRNAs Down-Regulate NRF2/GSH/GSSG to Reduce Oxidative Stress Damage

Nrf2 is an important component of an endogenous antioxidant system that translocates to the nucleus and activates gene expression of proteins associated with oxidative stress (91–93). Madhusudhanan et al. (94) showed that miR144, miR153, miR27a, and miR142-5p target the Nrf2 3′ UTR and inhibit endogenous Nrf2 mRNA, thereby down-regulating Nrf2 expression. In addition, overexpression of miR144, miR153, miR27a, and miR142-5p decrease the GSH/GSSG ratio and cellular ROS levels to reduce the damage caused by oxidative stress in neuronal cells.

### ncRNAs Which Act on a Particular Disease

#### LncRNA WT1-AS Down-Regulates WT1 to Inhibit miR-375/SIX4 and Reduce Oxidative Stress (AD)

In the AD model of Wang et al. (Aβ-treated SH-SY5Y cells), lncRNA WT1-AS expression was significantly reduced, but SOD was significantly increased after overexpression of lncRNA WT1-AS. This process may have occurred by reducing the expression of WT1 and suppressing miR-375. When neurons overexpress miR-375, SIX4 is significantly inhibited and tau protein phosphorylation is significantly increased, promoting oxidative stress and apoptosis. This implies that WT1-AS inhibits oxidative stress and apoptosis by regulating the transcription factor WT1 to suppress the miR-375/SIX4 axis (95).

#### LncRNA-p21 Competes With miR-1277-5p/miR625 to Increase Oxidative Stress (PD)

In the study of PD mouse models, Xu et al. (96) found that lncRNA-p21 expression was significantly increased and promoted apoptosis. It was found that lncRNA-p21 could directly bind and inhibit the activity of miR-1277-5p, then regulate the expression of the α-synaptic nuclear protein (α-SYN). α-SYN attenuated the ability of the neuron to fight oxidative stress in PD and increase apoptosis. This also meant that the impaired cellular oxidative stress in PD patients could be improved by regulating the lncRNA-p21/miR-1277-5p/α-SYN axis.

Meanwhile, Ding XM et al. found that in the SH-SY5Y cell model of PD, lncRNA-p21 attenuated neuronal oxidative stress by increasing SOD activity. P21, down-regulated miR-625 expression while up-regulating TRPM2 in neurons, which in turn increased neuronal injury (97).

#### LncRNA XIST Competes With miR-132 to Up-Regulate PTEN/FOXO3a and Increases Oxidative Stress (AD)

Wong HK et al. suggested that blocking miR-132/212 in neurons activated P300, and FOXO3a, which triggered the cascade response at the transcription factor FOXO3a and up-regulated death signals of BIM and caspase-3 to cause apoptosis. At the same time, the inhibited miR-132/212 suppressed the PI3K/AKT pathway by activating PTEN, which downregulated FOXO3a phosphorylation and further activated FOXO3a to cause cascade responses. In AD, down-regulation of miR132/212 expression in neurons up-regulates the levels of the target proteins PTEN and FOXO3a protein, then increases neuronal oxidative stress (98). In addition, Wang X. et al. found that lncRNA XIST expression was significantly elevated in the rat AD model induced by Aβ25-35, increasing neuronal oxidative stress and apoptosis. LncRNA XIST has six miR-132 binding sites and negatively regulates miR-132 expression by binding to the complementary miR-132 region. In contrast, knockdown of lncRNA XIST results in increased miR-132 expression and attenuated neuronal oxidative stress and apoptosis (99).

#### LncRNA SOX21-AS1 Inhibits FZD/Wnt Pathway to Promote Oxidative Stress (AD)

In the study of the AD mouse model, Zhang L. et al. found that MDA, OH, and lncRNA-SOX21-AS1 expression was significantly up-regulated in AD mice, accompanied by decreased levels of SOD, CAT, GSH-Px, FZD3 / 5, β-linked protein, etc. In contrast, silencing of SOX21-AS1 resulted in the opposite outcome and significantly reduced neuronal apoptosis in mice. Overall, these studies suggest that lncRNA SOX21-AS1 can stimulate neuronal oxidative stress associated with AD through FZD3/5 down-regulation and inhibition of the Wnt signaling pathway (100).

### Miscellaneous

Besides, some ncRNAs modulate neuroinflammation by regulating oxidative stress and mitochondrial dysfunction. For example, in PD, miR-27a/b negatively regulates PINK1 (101), miR-141-3p down-regulates NOS1/SIRT1 to promote mitochondrial dysfunction (102), miR-376a negatively regulates PGC1α, TFAM, etc., to promote oxidative stress (103), and miR-7/153 reduces oxidative stress by decreasing α-SYN expression (102). In AD, miR-23b activates the Akt/GSK-3β pathway to down-regulate p-tau (104), miR-9-5p activates Nrf2/ Keap1 to counteract oxidative stress (105), and miR-125b promotes Tau1 phosphorylation to promote oxidative stress (106). Regarding mitochondrial dysfunction and neuroinflammation, miR-195 can protect neurons by inhibiting JNK signaling, protecting oxygen from increasing, and mitochondrial dysfunction (107). Wang et al., found that miR-20a-5p levels were elevated in HT22 cells, and MiR-20a-5p played a neuroprotective role by targeting the IRF9/NF-κ B axis, inhibiting neuroinflammation, mitochondrial dysfunction, and oxidative stress response (108). In addition, miRNAs such as miRNA-92a-3P (109), 505-5p (110), and miR-34b/c (111) may also be involved in the interaction of mitochondrial dysfunction and neuroinflammation.

## Inflammatory Mediators

It is well-known that inflammatory mediators, as key components of the immune system, play an important role in the immune system of the body. Inflammatory mediators are also frequently seen in neurodegenerative diseases (3), and inflammatory mediators such as IL-1 (112), TNF-α (113), and IL-6 (114), have been reported to be involved in chronic neuroinflammatory processes in Alzheimer's disease and Parkinson's disease, which aggravate neuronal damage. Recent studies have given some pointers on how to regulate the expression of inflammatory mediators through ncRNAs and inhibit chronic neuroinflammation in neurodegenerative diseases.

### ncRNAs Which Act on a Variety of Neurodegenerative Diseases

#### LncRNA MALAT1

It was shown that the mRNA and protein expression of inflammatory mediators such as IL-6 and TNF-α were significantly reduced and IL-10 levels were increased in AD models with lncRNA MALAT1 overexpression, suppressing neuroinflammation. Knockdown of lncRNA-MALAT1 increases IL-6 and TNF-α levels and decreases IL-10 levels thus increasing neuroinflammation (115). In addition, miR-125b increases the expression of PTGS2 and CDK5, which promotes neuroinflammation and accelerates the process of AD. LncRNA MALAT1 may reduce neuroinflammatory damage in AD by down-regulating miR-125b expression (115).

However, in PD neuroinflammation-related studies, pro-inflammatory factors such as TNF-α, IL-1β, and IL-18 are significantly up-regulated in LPS/ATP-treated BV2 (a mouse microglial cell line), while knockdown of MALAT1 by sh-MALAT1 reverses the increased expression of these cytokines, suggesting that lncRNA MALAT1 can promote the occurrence of the inflammatory response by up-regulating inflammatory mediator expression (32).

Why the same lncRNA presents two opposite phenomena of pro-inflammation and anti-inflammation in AD and PD models, and the neuroinflammation-related mechanisms of both diseases remain to be investigated.

#### miR-132

In the qRT-PCR testing of brain tissues from PD patients vs. healthy controls, miR-132-3p expression was found to be 1.45-fold up-regulated in PD patients. In the MTPT-induced PD mouse model, after knocking down miR-132-3p, the inflammatory cytokine content was measured and inflammatory mediators such as IL-1β, TNF-α, and IL-6, were found to be significantly decreased. In contrast, miR-132-3p overexpression led to an increase in inflammatory mediator content (116).

In addition to this, miR-132 was observed to down-regulate the expression levels of TNF-α, IL-6, NO, and IL-1β in AD mice, possibly through the inhibition of HMGA2 expression (117).

### ncRNAs Which Act on a Particular Disease

#### LncRNA-GAS5 Targets miR-223-3p and Promotes Up-Regulation of Inflammatory Mediator Expression (PD)

In a study of PD-related neuroinflammation, inflammatory factor levels were measured by qRT-PCR testing, and GAS5 was positively correlated with levels of IL-1β, IL-6, and TNF-α, while miR-223-3p was negatively correlated with IL-1β, IL-6 and TNF-α (34). In subsequent experiments, knockdown of GAS5 significantly increased miR-223-3p expression, and inflammatory cytokines within IL-1β, IL-6, and TNF-α were significantly decreased (34), leading to the conclusion that GAS5 may exacerbate neuroinflammation by up-regulating related pro-inflammatory factors, while miR-223-3p had the opposite effect.

#### LncRNA-ANRIL Targets miR-125a and Promotes Up-Regulation of Inflammatory Mediator Expression (AD)

LncRNA-ANRIL targets bind to miR-125a and down-regulate its expression. By knocking down lncRNA-ANRIL, the expression of inflammatory mediators IL-1β, IL-6, and IL-17 is reduced, while knocking down miR-125 reversed this phenomenon. The researchers speculated that lncRNA-ANRIL promotes the expression of inflammatory mediators by down-regulating miR-125a (118). Taken together, lncRNA-ANRIL may exacerbate neuroinflammation by up-regulating related pro-inflammatory factors, while miR-125a had the opposite effect.

### Miscellaneous

In AD, miR-155 (119), miR-128 (120), miR-129-5p (121), and miR-485-3p (122) were reported to up-regulate the expression of inflammatory mediators such as IL-6, and TNF-α, to aggravate neuroinflammatory injury in AD, while lncRNA-MEG3 (43) down-regulated the corresponding inflammatory mediators in the hippocampus thereby reducing neuroinflammation. In contrast, lncRNA HOXA11-AS, which was studied in a previous paper, can up-regulate the proportion of M1-type microglia involved in the neuroinflammatory response and aggravate neuroinflammatory injury in PD by up-regulating IL-1β, IL-18, IL-6 and TNF-α (38).

## The Overall Synergy of the Four Mechanisms

**Figure 4:**
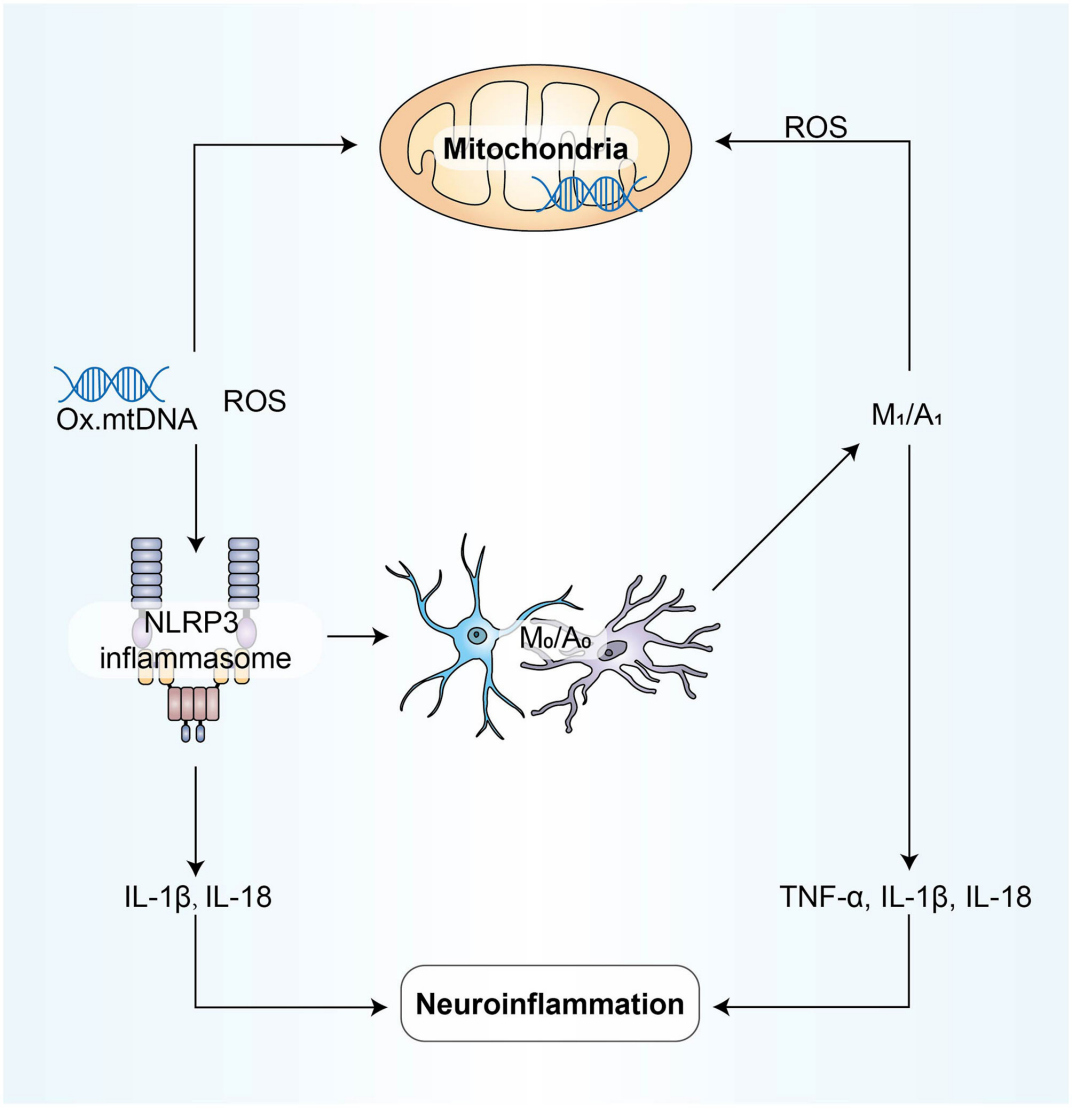
NLRP3 activation is involved in disrupting the pro-inflammatory and anti-inflammatory balance between microglia and astrocytes; M1/A1 type cells produce and release ROS and inflammatory factors, aggravating oxidative stress and mitochondrial dysfunction; due to mitochondrial dysfunction, mtROS produced by mitochondrial respiratory chain inhibition can activate NLRP3. Inflammatory mediators play a bridging role in these processes above, and all mechanisms eventually participate in neuroinflammation directly through the form of inflammatory mediators.

In previous studies, we can find that some ncRNAs affect neuroinflammation through different mechanisms, suggesting that our four mechanisms are not independent of each other. They coordinate and influence each other and participate in the process of neuroinflammation (Figure 4).

## Conclusions and Future Directions

Neurodegenerative diseases place heavy economic and medical pressure on patients and society, but the current treatment programs are not satisfactory. At present, the treatment of PD and AD are facing similar problems: taking symptomatic drugs cannot reverse the degenerative process, the symptoms of patients still deteriorate gradually with time and the demand for symptomatic drugs tends to increase (123).

With the progress in the study of the mechanism of neurodegenerative diseases and neuroinflammation, researchers have shown that neuroinflammation plays an important role in neurodegenerative diseases (124). Through four major inflammatory mechanisms: imbalance of the pro-inflammatory and anti-inflammatory ratio of microglia and astrocytes, high expression of NLRP3 inflammatory bodies, large secretion of inflammatory mediators, and oxidative stress and mitochondrial dysfunction, neuroinflammation is widely involved in the occurrence and development of neurodegenerative diseases.

Recent studies have revealed that some substances with anti-inflammatory effects have shown their influences on neurodegenerative diseases, such as sulforaphane (SFN) (125), caffeine (126), curcumin (127), N-acetylcysteine (128), and so on, proving that the idea of anti-inflammatory treatment has certain feasibility.

In recent years, the application of ncRNAs in the treatment of diseases in various fields has developed rapidly and has achieved significant success. A variety of RNA therapeutic drugs have been developed, including ASO, SMIRs, AMO, miR sponge, and miR mimics for miRNAs (129). At present, ncRNAs targeted therapeutic drugs for a variety of organ system diseases have entered the clinical trial stage, such as RG-125, a miR103/107 antagonist for type 2 diabetes and non-alcoholic fatty liver (130). In addition, miRNAs therapy has been widely used in the field of anticancer therapy and achieved some results (129).

In the field of neurodegenerative diseases, it has achieved a significant curative effect on disease models such as PD (38), MS (47), and AD (43) through the regulation of ncRNAs expression in animal disease models to regulate neuroinflammation. However, we still see a series of problems that need to be overcome in miRNAs and lncRNAs targeted therapy for neurodegenerative diseases, like, for instance, how to deliver the therapeutic agent to the target tissue through the blood-brain barrier, or how to overcome the degradation of nuclease and the ineffective endocytosis of target cells and their clearance in the body. Attention should also be paid to the unexpected adverse effects of miRNAs and the subsequent adverse consequences, such as the regulation of a particular miRNA may be beneficial to one cell type and harmful to another (129).

In the experiments related to the regulation of neuroinflammation by ncRNAs, we observed the gratifying changes produced by knocking down the pro-inflammatory ncRNAs in the animal model of neurodegenerative diseases, including the recovery of cognitive and motor functions and the regression of neuroinflammation. Therapies involving miRNAs/lncRNAs may break through the traditional symptomatic treatment and the limitation of the specific disease. Some ncRNAs targets that act on a variety of neurodegenerative diseases provide the possibility for combined therapy of disease. Moreover, many studies using miRNA and lncRNA as biomarkers to assist in the diagnosis and prognosis of neurodegenerative diseases are still ongoing such as “Study on Novel Peripheral Blood Diagnostic Biomarkers for MCI Due to Alzheimer's Disease,” “Study of miRNA Expression Pattern as Diagnostic and Prognostic Biomarker in Amyotrophic Lateral Sclerosis,” and “Protein and microRNA Markers for Early Detection of Alzheimer's Disease.”

## Author Contributions

HJ: conceptualization of the study, project supervision, critical discussion, and manuscript drafting. YZ and JY: literature review, critical discussion, and manuscript drafting. YS: manuscript drafting. ZL and WX: outline design and manuscript revision. BX: financial support. All the authors approved the publication of the manuscript. All authors contributed to the article and approved the submitted version.

## Funding

This study was supported by grants from the National Natural Science Foundation of China (grant number: 81974206) and the Innovative Construction Foundation of Hunan Province (grant number: 2021SK4001).

## Conflict of Interest

The authors declare that the research was conducted in the absence of any commercial or financial relationships that could be construed as a potential conflict of interest.

## Publisher's Note

All claims expressed in this article are solely those of the authors and do not necessarily represent those of their affiliated organizations, or those of the publisher, the editors and the reviewers. Any product that may be evaluated in this article, or claim that may be made by its manufacturer, is not guaranteed or endorsed by the publisher.
